# Infrastructure challenges to doing health research “where populations with the most disease live” in Covid times—a response to Rai et al. (2021)

**DOI:** 10.1186/s12874-022-01737-z

**Published:** 2022-10-08

**Authors:** Jennifer MacLellan, Joanne Turnbull, Catherine Pope

**Affiliations:** 1 Nuffield Department of Primary Care Health Sciences, University of Oxford, Radcliffe Observatory Quarter, Woodstock Road, Oxford, OX2 6GG United Kingdom; 2 University of Southampton Health Sciences, Building 67 Highfield, Southampton, SO17 1BJ United Kingdom

**Keywords:** Barriers to research, Case study, Health need

## Abstract

**Background:**

The failure of randomised controlled trials to adequately reflect areas of highest health need have been repeatedly highlighted. This has implications for the validity and generalisability of findings, for equity and efficiency, but also for research capacity-building. Rai et al. (BMC Med Res Methodol 21:80, 2021) recently argued that the poor alignment between UK clinical research activity (specifically multi-centre RCTs) and local prevalence of disease was, in part, the outcome of behaviour and decision-making by Chief Investigators involved in trial research. They argued that a shift in research culture was needed. Following our recent multi-site mixed methods evaluative study about NHS 111 online we identify some of the additional structural barriers to delivering health research “where populations with the most disease live”, accounting for the Covid-19 disruption to processes and delivery.

**Methods:**

The NHS 111 study used a mixed-method research design, including interviews with healthcare staff and stakeholders within the primary, urgent and emergency health care system, and a survey of users and potential users of the NHS 111 online service. This paper draws on data collated by the research team during site identification and selection, as we followed an action research cycle of planning, action, observation and reflection. The process results were discussed among the authors, and grouped into the two themes presented.

**Results:**

We approached 22 primary and secondary care sites across England, successfully recruiting half of these. Time from initial approach to first participant recruitment in successful sites ranged from one to ten months. This paper describes frontline bureaucratic barriers to research delivery and recruitment in the local Clinical Research Network system and secondary care sites carrying large research portfolios, alongside the adaptive practices of research practitioners that mitigate these.

**Conclusions:**

This paper augments the recommendations of Rai et al., describing delays encountered during the COVID-19 pandemic, and suggesting in addition to cultural change, it may be additionally important to dismantle infrastructural barriers and improve support to research teams so they can conduct health research “where populations with the most disease live”.

## Introduction

Clinical and health services research provide the evidence base for improving healthcare and services and are vital to reducing health inequalities. Yet the failure of randomised controlled trials to adequately recruit from areas of highest health need has been repeatedly highlighted [1–4]. Bower et al. [5] noted especially low recruitment in areas with higher rates of long term conditions and mental health conditions and argued that at least 12% of current recruitment activity would need to be redistributed to align with disease prevalence. Improving patients’ access to research is actively promoted [6], and is a core plank of the UK National Institute for Health Research strategy, enshrined in documents such as Going the Extra Mile [7], and Best Research for Best Health [8]. However, recruiting patients and/or healthcare staff located in areas of high health need is not straightforward. Potential participants in areas of intersectional poverty and multiple comorbidities face challenges to participation that could include awareness of research, time available to participate, understanding of study benefits, access to resources to support participation (e.g. health literacy, phone data for interviews or surveys) [9]. NHS staff working in these areas have correspondingly high clinical workloads, and may lack access to adequate research capacity and support [10].

Rai et al. [11] interviewed Chief Investigators (CIs) of multi-centre, portfolio research trials (that have Clinical Research Network (CRN) research delivery support) to explore how research sites were selected, and to examine how CI behaviour might contribute to the lack of research participant recruitment in areas of high health need. Their study suggested that while prevalence of disease was a consideration in site selection, these highly successful CIs favoured the selection of research sites within their professional networks that by default were research centres with a strong research history and infrastructure in place. Such sites could support the timely, successful delivery of study objectives, thereby enhancing CIs professional reputation and their success in securing future research funding. CIs interviewed by Rai et al. believed (rather than reporting direct experience) that engaging research sites in areas of high need would require more time and effort to build capacity among less experienced research partners. While Rai et al. noted those CIs who ‘broke the mould’ and actively identified and nurtured new sites were often successful in meeting recruitment targets and delivering their studies to deadline, the widely-held perception that this was more difficult, or risky, was a significant deterrent to many CIs.

## Methods

### Our study

The protocol (https:njl-admin.nihr.ac.uk/document/download/2035910.pdf) for our NIHR funded study about NHS 111 Online made an explicit commitment to try to access sites in areas of high health need to participate in our research. The NHS 111 Online study was not a multi-centre randomised controlled trial, but used a mixed-method case study design to investigate an online urgent care triage and signpost system of high relevance to areas of high health need due to their corresponding high emergency care use [12]. The study involved a survey of users and potential users of the NHS 111 online urgent care triage and assessment service, and qualitative interviews with health care professionals, staff and stakeholders working in or with primary, urgent and emergency health care services across England.

### Site recruitment using the Action Research Cycle

**Fig. 1:**
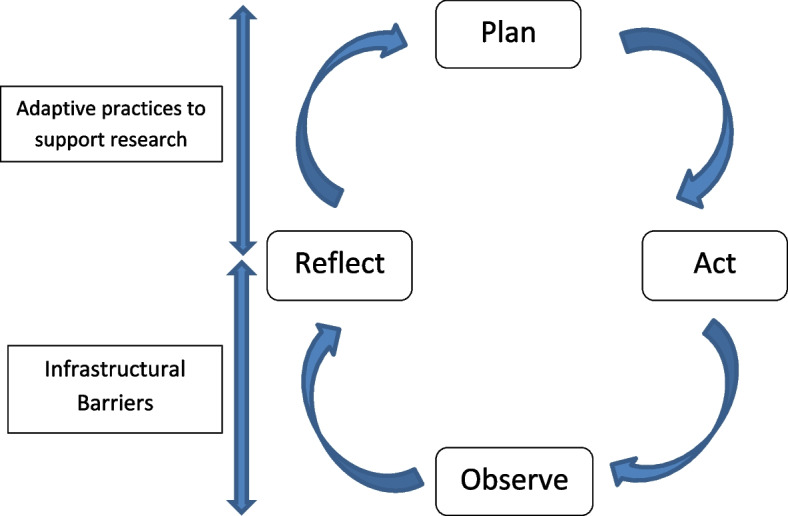
The Action Research Cycle

Recruitment of study sites was informed by principles of Action Research and attempted to balance problem-solving actions with data-driven collaborative analysis to understand underlying causes and enable change [13]. This understanding fed into a revised site recruitment plan and a restart of the cycle (Fig. 1. The Action Research Cycle). Our commitment to conducting research in areas of high health need meant identifying and working with less research central sites, for example the district general hospital ED rather than the central trauma centre or primary care services with specific practice profiles. This approach stimulated collaborations with new organisations across England. These challenges were overlain by the unexpected, additional challenge of conducting our research in the period during which Covid-19 spread across the UK, resulting in pauses to our research as ‘urgent public health’ research was prioritised.

Data collection began in October 2020 and ran until July 2021, spanning two national lockdowns (November 2020, January 2021). Non-Covid-19 research activity was postponed in our secondary care sites during these times to prioritise resources, however we continued data collection without restriction in primary and urgent care. Following moves to remote working as a result of the Covid-19 pandemic, interviews were conducted over the telephone and the survey completed online, (or on a computer tablet in urgent or emergency care services), facilitated by a research nurse.

We conducted a geographical review of areas of high health need in England, noting the frequent intersection of health needs with deprivation and higher ethnic minority proportion in the population. Our site selection was informed by area deprivation scores, socio-demographic characteristics of the population, including age profiles and ethnic composition due to links with high levels of comorbidity, and service provision, health literacy, and help seeking resource constraints [14, 15]. We used the Index of multiple deprivation (IMD) to map areas of greatest deprivation in England due to their known association with high health need [16, 17]. We also made particular efforts through the CRN to engage with GP practices, district hospitals and community Trusts that served more ethnically diverse populations and by consequence carried a smaller study portfolio. We mapped literacy scores from the National Literacy Trust as a proxy indicator of potential barriers for public access and use of digital health technologies. Together these scores and indicators helped us to target our site selection to primary and secondary care sites in coastal areas of Kent, Sussex and the North West, areas of Yorkshire, Humberside, Shropshire, Berkshire, Thames Valley, Bedfordshire and Wessex that contain mixed urban/rural populations, and known pockets of deprivation, as well as the more central research sites of major NHS Teaching hospitals of Birmingham, Cambridge, the South Coast and London.

The lead researcher (JM) liaised with 8 NIHR Clinical Research Networks (CRNs) across England to support recruitment of case study sites, and in particular to help identify services with a limited research portfolio within these target areas. Prior to the pandemic lockdowns the principal investigator (CP) also attended a regional CRN urgent and emergency care network meeting and visited general practices in this area to present the study and encourage participation. The study was registered on the national NIHR portfolio, thus site recruitment would be credited to the site’s CRN account and contributes to future investment in research infrastructure and resources of the region. The study team contacted regional CRN teams responsible for primary or secondary care research. For primary care sites, the CRN reviewed the study governance and approval documentation before sending out an ‘expression of interest’ email to potential primary care/urgent care sites. The clinical leads replied to the CRN expressing their interest to participate as a site. The CRN passed their details to the study team who sent the research pack containing full details of the study requirements and processes. Once the site confirmed willingness and ability to participate, contracts were exchanged and they became an open and recruiting site of the study. The process was slightly different for secondary care sites. The study team contacted the generic CRN study support email with their request. In some cases the CRN liaised with the emergency department (ED) research team, in others the study team were given the details of the team lead to discuss capacity and interest in potential participation as a research site. The ED research team lead returned an expression of interest to participate as a site to the study team after discussion with their clinical team. The research pack containing full details of the study requirements and processes was then sent to the ‘research and development’ department of the hospital for review. Once contracts were exchanged and confirmation of capacity and capability was issued, they became a recruiting site of the study.

This paper reports our experience of trying to do health research “where populations with the most disease live”. We identify some of the structural barriers faced by health researchers, accounting for the Covid-19 disruption to processes and delivery. Throughout the recruitment process, the research team reflected on progress and engaged in collaborative problem solving in bi-weekly meetings, working with CRN teams and contacts from professional networks to diversify recruitment and support the NIHR’s goal of bringing applied research closer to communities that are often under-served by research. We met as a research team to review progress, updating timelines and logs of interactions with sites to identify problems and issues.

## Results

Reflection and analysis of our observations identified two themes impacting the effort to deliver equitable health research. These were: infrastructural barriers to research and adaptive practices implemented by the research team and research nurses in practice to mitigate these.

### Infrastructural barriers to research

This theme presents the actions and observations of our site recruitment experiences as mapped to the Action Research Cycle. Table 1 shows the timeline of site set up from initial approach to study approval. In primary care, CRNs cover a large number of practices in a region. Site engagement is dependent on the CRN governance review and then their relationship with general practices that meet the study criteria. The CRN primary care teams reviewed the ethics documents that had been approved prior to commencement by the Health Research Agency (HRA). In six sites this took several months (D, J, N, Q, U, V), delaying the granting of permission for the CRN delivery team to contact local sites for participation. This impacted the timeline of the study as no contact was made with potential sites until this review was complete. An exception was one CRN team who accepted all the HRA governance documents and sent out expression of interest to sites within a few days (T). This CRN used their network of GP champions to commence recruitment quickly and to cascade the study out to colleagues in areas of high health need.

**Table 1:**
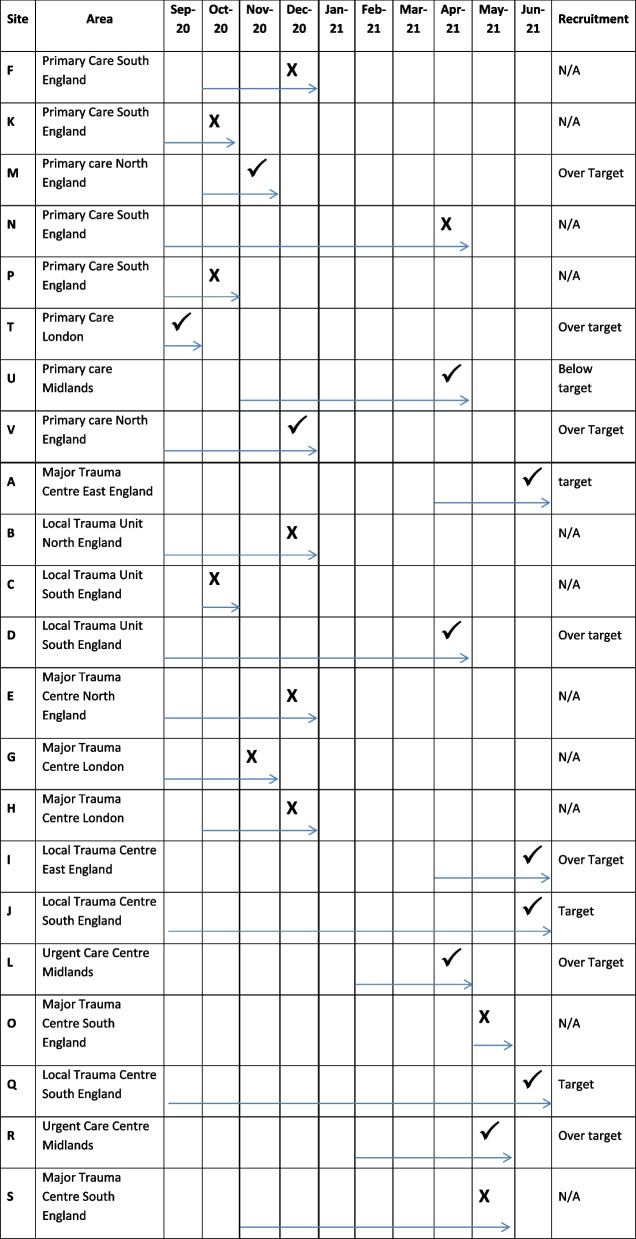
Timeline – site approach to study approval

Arrows indicate the timeframe of active consideration. Tick indicates approval granted

Liaising with some CRNs to approach general practices with a limited research portfolio in areas of deprivation or with ethnically diverse populations often required several conversations by phone or email. Most CRNs had stronger links with practitioners in previously research active practices, who tended also to be those with predominantly white and middle class patient lists. Access to primary care sites was highly dependent on the activity and commitment of the CRN link person. In one case, when no practices expressed an interest to join the study after two newsletter communiques (F) discussion of further strategies for engagement failed to identify either a research champion in this CRN, or any engagement events that could be accessed by the research team. In another CRN, the study team were sent a list of practice addresses obtained from a Google search and were advised to make contact directly with these, as the CRN did not have any working links with these practices (U).

Links between the CRN and local secondary care governance was often patchy and this too impacted on progress. The study team encountered an under developed website without governance team contact details (I), unsupported or unavailable governance staff (J, Q) and a lack of available research staff to support the study (B – no research staff assigned to the site, E – single research nurse pulled in to practice due to clinical caseload demands of Covid-19). Governance processes were protracted and delayed (D, J, N, Q, U, S), despite only three sites shutting down non-urgent public health research during the national lockdowns to prioritise Covid-19 studies (D, J, Q), while two sites declined participation due to a heavy research workload (G, O, S), one reported:

“…they [ED research team] are not in the position to take this study forward at this time. The team are already facilitating a number of high recruiting studies” (O).

These delays ranged from two to 10 months.

Some of the smaller secondary care sites appeared to have faster governance processes, averaging three months from contact to first recruitment, and this may have been because they had less involvement in Covid-19 related research. However supporting a smaller research portfolio meant there were less CRN funded research nurses (because these are linked to recruitment accrual) to deliver the survey component of the study; thus two emergency department sites did not open because there was no Research Nurse support (B, E). In a third emergency department site the Research practitioner was allocated one day per week to support the study which meant that it took longer to achieve target recruitment for the survey (I).

### Adaptive practices to support research

The research team adapted their processes to support site participation following bi-weekly team reflection on experience and revising of the plan as per the Action Research Cycle. They communicated closely with the primary care CRN teams to engage sites in target areas, presenting the study at network meetings (T) and writing communiques about the study for local CRN newsletters (F, R). Offering flexibility for interview format and timing by the research team allowed NHS staff, in particular clinicians, to balance interview participation with their workloads. This required the researchers being available at short notice, conducting remote interviews while staff travelled and being available at the weekend and in the evenings. For the public facing survey we found collating and reporting weekly recruitment figures to sites using the CRN reporting format, rather than using monthly or end of study reports kept recruitment on track. With permission from HRA, rather than simply paying a block fee to cover ‘administration’ for CRN delivery of the survey, we linked the survey incentive payment directly to the site recruitment numbers. This, combined with the weekly accrual reports encouraged CRN promotion and support of the survey.

Rai et al. [11] observed research fatigue in sites delivering a large research portfolio with the number of competing studies a barrier to recruitment. Our study found a similar context, exacerbated by the burden of Covid-19 research and inpatient case load of major NHS teaching hospitals (A, G, H, O, S). We found that once governance was complete, sites with a smaller portfolio consistently recruited above target. These sites reported that the need to achieve ‘recruitment numbers’ was a motivator (D, I, L, M, R, T, V), to survey recruitment. Some of these sites were innovative in their use of resources to maximise recruitment. One site sent their CRN nurse to each facility in their region to present the study and increase target recruitment in the public survey and staff interviews (R). Another site arranged to deliver the survey component of the study alongside a Covid-19 vaccine clinic, working with the local CRN nurse to create QR codes for the survey and laminated information materials about the study (L). One emergency department site temporarily moved a research nurse from another department to support the study explaining:

“ we do not have a research nurse in A&E but one of our research practitioners has one day/week to support the study.”

Some secondary care sites located spare computer tablets to enable survey data collection rather than putting the study team tablet through local facility Portable Appliance Testing (PAT) and causing further delays. These responsive and pro-active measures of the local CRN nurses enabled the study to achieve good survey responses even in sites that had little previous experience of participating in this kind of research study.

## Discussion

It took significantly more time and effort to identify and recruit participants for the public survey and staff interviews in areas identified as having high health needs and a small research portfolio. Some of the challenges encountered were in part related to the impact of COVID-19 both directly on health services that we wanted to study, and on research infrastructure support which was depleted due to prioritisation of COVID-19 research and impact of staff sickness. The CRN infrastructure, tasked with supporting set up of research in the NHS, proved challenging for the study team due to regional differences in requirement, operating procedures and communication which persist outside ‘COVID times’. Despite the introduction of the streamlined governance processes for national HRA and ethics approvals, there were considerable bureaucratic delays to local governance processes and research initiation. This concern has been expressed elsewhere and is under investigation in the Independent Review of Research Bureaucracy [18]. There was variation in how the CRNs approached supporting this study, and, crucially, the time they took to approval. The CRN acted as gatekeeper to recruitment of general practices but in some areas CRN coordinating staff had few connections to, and little experience of, working with general practices or other primary care sites (Minor injury units, urgent care centres, emergency dentists) with a small research portfolio. For secondary care, site set up could also be initiated directly via NHS Trust research offices, but here too there were some significant delays to local governance approvals. In contrast, the CRN practitioners on the ground recruiting participants were flexible, adaptable and motivated to work closely with the research team to deliver the study requirements.

Our study sought to recruit staff, stakeholders, patients and the public in relation to use of a non-disease specific service, and the design and data collection methods were less familiar for CRN governance staff than a treatment intervention design, which may have contributed to approval delays. Recruitment of sites that could target populations most at risk of healthcare inequalities was less well supported by some CRNs, whose resources were focused in secondary care and with particular GP practice profiles. In some sites there was a research nurse or other clinician who took responsibility for research delivery and processes, and this was beneficial to widening the opportunities for staff and the public to participate in our study. Staff workloads in sites demanded flexibility from the study team to maximise opportunities for data collection, as has been reported elsewhere [19]. The provision of weekly accrual data combined with targeted incentive payment appeared to motivate survey recruitment.

### Strengths and limitations

Our study was observational and low risk, and did not demand detailed clinical knowledge, specific disease profiles, trial processes or clinical monitoring from the delivery practitioners. However, local governance processes and support provided by NIHR CRN infrastructure was highly variable and led to delays and failures to recruit. The study and surrounding infrastructure was undoubtedly impacted by the Covid-19 pandemic, which we have accounted for in our analysis. However, governance review and site identification took place in periods when non-Covid related research was meant to be supported. Our experience has uncovered inconsistent processes across the network and a historical concentration of focus on privileged areas of the health service and profiles of the population in many areas. The barriers we have identified to conducting research “where populations with the most disease live” are not new, nor are they confined to the impact of the pandemic.

### Implication of our findings

We agree with Rai et al. that Chief Investigators and researchers more generally have much to do in ensuring that health and care research better represents the populations who use care services. However, in addition to this much needed cultural change, there is a need to examine the bureaucratic and infrastructural barriers to delivering equitable research. Given the significant costs allocated to research support infrastructure it seems timely to consider how these barriers can be removed. International collaboration is essential for the sharing of skills and knowledge, most pertinently in the recent Covid-19 pandemic that did not respect borders. The temporary streamlining of bureaucratic processes within the UK and between international partners, kept participants safe while delivering a swift research response that identified effective treatments and vaccines for the population in record time. Learning from the example of the pandemic response could impact bureaucratic processes across research to increase equity and the timely impact of findings.

## Conclusion

This paper has outlined some important infrastructure challenges to delivering health care research in places where there is greatest health need. Working with sites that historically have delivered fewer research studies can support better development of their infrastructure and capacity through training and support by the study team. Cascade networks in primary care such as the research champion network could increase engagement with practices in areas of high health need by sharing this model across the network. Extension of funding for CRN staff to support research in practices and community facilities (such as minor injury units) with a heavy clinical workload could support research participation from a representative population while developing research capacity and interest. Transparency in academic researcher job descriptions would ensure a commitment to adaptive ways of working that align with the configuration of clinical practice. An accountable feedback mechanism from researchers to the CRN regarding barriers to study delivery, as well as recognition of excellent performance, should be formalised.

## Data Availability

Data sharing is not applicable to this article as no datasets were generated or analysed during the current study.
